# mUzima Mobile Electronic Health Record (EHR) System: Development and Implementation at Scale

**DOI:** 10.2196/26381

**Published:** 2021-12-14

**Authors:** Martin Chieng Were, Simon Savai, Benard Mokaya, Samuel Mbugua, Nyoman Ribeka, Preetam Cholli, Ada Yeung

**Affiliations:** 1 Department of Biomedical Informatics Vanderbilt University Medical Center Nashville, TN United States; 2 Institute of Biomedical Informatics Moi University Eldoret Kenya; 3 Digital Impact Alliance Washington, DC United States

**Keywords:** mobile health, electronic medical records, developing countries, digital divide, digital health, global health

## Abstract

**Background:**

The predominant implementation paradigm of electronic health record (EHR) systems in low- and middle-income countries (LMICs) relies on standalone system installations at facilities. This implementation approach exacerbates the digital divide, with facilities in areas with inadequate electrical and network infrastructure often left behind. Mobile health (mHealth) technologies have been implemented to extend the reach of digital health, but these systems largely add to the problem of siloed patient data, with few seamlessly interoperating with the EHR systems that are now scaled nationally in many LMICs. Robust mHealth applications that effectively extend EHR systems are needed to improve access, improve quality of care, and ameliorate the digital divide.

**Objective:**

We report on the development and scaled implementation of *mUzima*, an mHealth extension of the most broadly deployed EHR system in LMICs (OpenMRS).

**Methods:**

The “Guidelines for reporting of health interventions using mobile phones: mobile (mHealth) evidence reporting assessment (mERA)” checklist was employed to report on the *mUzima* application. The World Health Organization (WHO) Principles for Digital Development framework was used as a secondary reference framework. Details of *mUzima*’s architecture, core features, functionalities, and its implementation status are provided to highlight elements that can be adapted in other systems.

**Results:**

*mUzima* is an open-source, highly configurable Android application with robust features including offline management, deduplication, relationship management, security, cohort management, and error resolution, among many others. *mUzima* allows providers with lower-end Android smartphones (version 4.4 and above) who work remotely to access historical patient data, collect new data, view media, leverage decision support, conduct store-and-forward teleconsultation, and geolocate clients. The application is supported by an active community of developers and users, with feature priorities vetted by the community. *mUzima* has been implemented nationally in Kenya, is widely used in Rwanda, and is gaining scale in Uganda and Mozambique. It is disease-agnostic, with current use cases in HIV, cancer, chronic disease, and COVID-19 management, among other conditions. *mUzima* meets all WHO’s Principles of Digital Development, and its scaled implementation success has led to its recognition as a digital global public good and its listing in the WHO Digital Health Atlas.

**Conclusions:**

Greater emphasis should be placed on mHealth applications that robustly extend reach of EHR systems within resource-limited settings, as opposed to siloed mHealth applications. This is particularly important given that health information exchange infrastructure is yet to mature in many LMICs. The *mUzima* application demonstrates how this can be done at scale, as evidenced by its adoption across multiple countries and for numerous care domains.

## Introduction

### Background

Low- and middle-income countries (LMICs) have, over the last decade, seen an exponential increase in the adoption of digital health solutions. Among the systems being actively implemented in these settings are electronic health record (EHR) systems. These are deployed to largely replace or supplement existing paper-based records, with the aims of improving quality of patient care and supporting the monitoring and evaluation of programs [1-3]. Several LMICs have gone beyond initial pilot EHR system implementations to large-scale rollout of these systems in government-run public facilities. Countries like Kenya, Uganda, Nigeria, and Mozambique now run nationally endorsed EHR systems that are deployed in hundreds to thousands of public health facilities across each country [4-7].

In most LMICs, national-level EHR system initiatives have largely been driven by the need to support HIV care and treatment, with significant funding coming from donor organizations such as the U.S. President’s Emergency Plan for AIDS Relief. EHR systems targeting HIV care largely focus on the HIV care continuum, which emphasizes (1) finding patients who are HIV-positive (through active screening approaches), (2) linking HIV-positive patients to care, (3) ensuring that patients are on appropriate treatment, and (4) actively following patients to retain them in care [8-10]. The continuum of care paradigm is widely applicable for numerous other chronic diseases and is employed within other EHR systems to support longitudinal care in LMICs.

### Approaches and Gaps in HIV EHR System Implementations Within LMICs

There are several core functionalities needed within EHR systems to support longitudinal care, key among them being the abilities to register patients, review historical patient information, and collect new data on patients. Features such as computerized decision support, order entry capabilities, and electronic prescribing are often incorporated at varying levels [11]. In many settings, retrospective entry of data is still employed, though efforts are underway to increase use of EHR systems at the point of care [12]. Point-of-care EHR systems still face the challenges of inadequate infrastructure, unreliable system uptime, cost, busy care settings, and provider discomfort with real-time entry of data while caring for patients.

The most widely employed modality of EHR system implementation in LMICs involves standalone EHR system implementations at individual facilities [13]. This implementation model entails installation of a local server, local area network, and end user terminals. The model relies on a dependable electrical supply and readily available information technology personnel [14]. Unfortunately, these infrastructure and personnel requirements are prohibitive in many LMIC settings, particularly in remote areas—exacerbating the “digital divide.” Facilities in areas with limited power, internet connectivity, and technical support are thus less likely to implement EHR systems [15]. Further, population health programs and community-based care services occurring outside of care facilities, such home-based HIV screening, testing, and patient tracing, are often poorly supported when access to EHR systems is limited to facilities.

To address some of the challenges and gaps with tethered EHRS that can only be accessed from within facilities, mobile health (mHealth) solutions are increasingly being adopted in LMICs [16,17]. Ideally, these mHealth systems should exchange data with the predominant EHR system, but a majority do not [18]. Instead, most mHealth systems often collect and transmit data to their own independent repositories that are separate from facility-based EHR systems—adding to the problem of siloed information for patients spread across multiple systems [19]. In the absence of interoperability with facility-based EHR systems, mHealth solutions cannot receive and display comprehensive historical patient and treatment information that are stored in the separate EHR systems, adversely affecting quality of care. Even when mHealth systems are able to share data with facility-based EHR systems, there is often lack of robust deduplication approaches for patient data, causing further challenges. Other common challenges observed with attempts to synergize mHealth solutions with facility-based EHR systems include (1) a lack of robust mechanisms to generate subsets of patients from EHR systems to be availed on mobile devices that have limited data storage capacities; (2) inefficient data synchronization, with mHealth applications requiring a complete wiping of existing data prior to new updates, resulting in increased expenses from use of paid internet data known as data bundles; (3) suboptimal mechanisms for updating or adding new forms, with some mHealth solutions requiring a new version of the application to be installed every time new forms are deployed; and (4) inability to handle forgotten log-in credentials that are aligned with EHR system credentials when providers are off-site.

With renewed emphasis on reaching the “last digital mile” while ensuring health information exchange with existing EHR systems, mHealth solutions are needed that can seamlessly interoperate with existing EHR systems in LMICs. This exchange is needed even in settings where health information exchange infrastructure is yet to mature [20]. In this paper, we present one such mobile application, *mUzima* [21], a UNICEF-recognized digital global public good [22], as a demonstration of a successful extension of existing national-level EHR systems in several LMICs, with the aim of increasing access and reach of digital technologies for providers and patients. We report on *mUzima*’s features and functionality, guided by the mHealth Evidence Reporting and Assessment (mERA) guidelines [23] and referencing the World Health Organization (WHO) Principles for Digital Development framework [24] where relevant. We also provide real-world examples of how the *mUzima* application has been scaled to support care across several countries and disease domains.

## Methods

The “Guidelines for reporting of health interventions using mobile phones: mobile (mHealth) evidence reporting assessment (mERA)” checklist was developed by the WHO mHealth Technical Evidence Review group to improve comprehensiveness and standardization of reporting of mHealth interventions [23]. The guidelines ensure that the reporting covers the (1) content of the mHealth intervention, (2) context within which the mHealth intervention is implemented, and (3) technical features of the intervention. These 3 components are encapsulated in the *mERA* checklist of 16 elements, namely (1) infrastructure (population level), (2) technology platform, (3) interoperability/health information system (HIS) context, (4) intervention delivery, (5) intervention content, (6) usability/content testing, (7) user feedback, (8) access of individual participants, (9) cost assessment, (10) adoption inputs/program entry, (11) limitations for delivery at scale, (12) contextual adaptability, (13) replicability, (14) data security, (15) compliance with national guidelines or regulatory statutes, and (16) fidelity of the intervention.

This paper describes in detail each element of the mERA checklist as relevant to the *mUzima* application. Special emphasis is placed on the technology platform (item #2) to highlight choices made and features of the application that can serve as a technical reference for readers. As the *mUzima* application is developed for a global context, additional considerations need to be given to its applicability in LMICs. To this end, we also integrate components of the Principles of Digital Development by the WHO, using this framework to further elucidate the various items in the mERA checklist [24]. The Principles are “designed to help integrate best practices into technology-enabled programs...and are derived from lessons learned through the use of information and communication technologies (ICTs) in development projects” [24]. The 9 principles include (1) design with the user; (2) understand the existing ecosystem; (3) design for scale; (4) build for sustainability; (5) be data driven; (6) use open standards, open data, open source, and open innovation; (7) reuse and improve; (8) address privacy and security; and (9) be collaborative. Reference to these principles extends the relevance of the reported work to the larger LMIC and global context.

## Results

‘*Uzima*’ is a Swahili word that means “life”—hence *mUzima’s* slogan “Mobile for Life” [21]. It is a provider-facing mHealth application for use by health care workers for direct patient care. *mUzima* has particular relevance to providers working outside of care facilities and those with unreliable connection to the EHR system. In the following sections, we provide details of the *mUzima* application in line with 16 mERA checklist items.

### Item 1: Infrastructure

**Figure 1:**
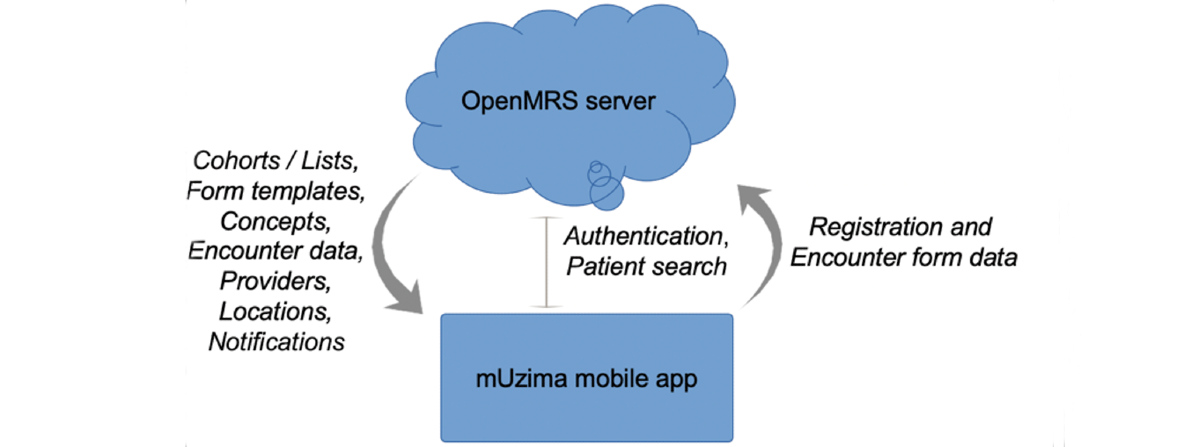
Interaction between *mUzima* and the OpenMRS electronic health record (EHR) system.

*mUzima* is a robust and adaptable Android-based mHealth platform that can seamlessly interoperate with the OpenMRS EHR system (Figure 1) [25,26]. The OpenMRS EHR system was chosen for the mHealth extension, as it is the most widely endorsed EHR system for national use by Ministries of Health (MoHs) across numerous LMICs. OpenMRS is currently in use in over 40 countries, with national deployments for HIV and tuberculosis care in Kenya, Mozambique, Nigeria, and Uganda, among others [27]. The hardware requirements for *mUzima* include an Android smartphone device and an OpenMRS EHR system instance. In Africa, over 85% of smartphones use the Android operating system [28]. *mUzima* was developed to work with Android versions 4.4 and above, as low-end Android smartphones predominate in LMICs. *mUzima* has robust offline functionality to address limited internet connectivity in many LMICs, allowing for health providers using *mUzima* to care for patients even when disconnected from the EHR system server. These infrastructure considerations align with the Digital Development Principles of “Understanding the Existing Ecosystem” by recognizing that power and internet connectivity challenges exist in the LMIC settings where *mUzima* is intended for use and that low-end Android smartphones predominate in these settings.

### Item 2: Technology Platform

#### Overview

**Figure 2:**
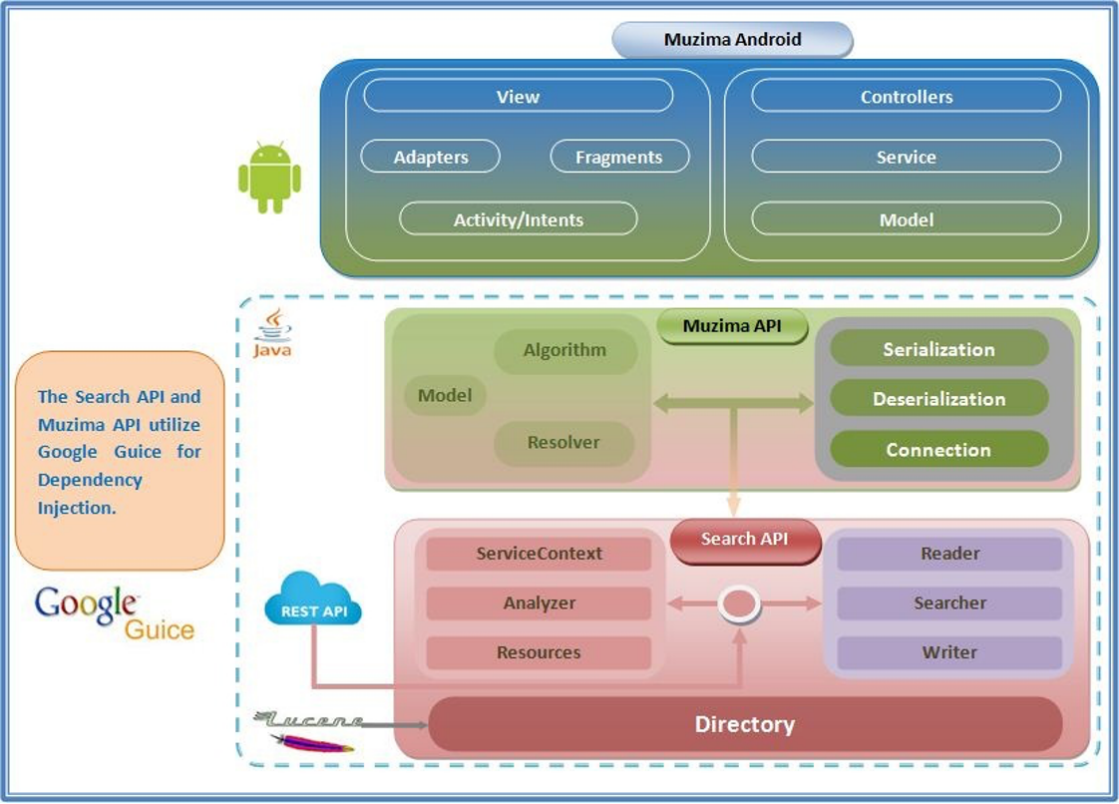
Architecture of the *mUzima* mobile health (mHealth) application. API: application programming interface; REST: representational state transfer.

*mUzima* was developed as an open-source application under the Mozilla Public License 2.0 license [29]. The application has a modular architecture that lends it a strength of simplicity while ensuring full functionalities of the installed modules. *mUzima* uses a stacked framework consisting largely of 3 layers (Figure 2), namely the (1) Search Application Programming Interface (API), (2) *mUzima* API, and (3) *mUzima* Android.

The Search API provides encrypted data storage based on a robust and flexible data model. This API utilizes Lucene, a Java-based, open-source, searching engine library, for indexing and searching [30]. Within the Search API are the service layer and a dependency injection layer using Guice [31] and the Indexer document repository [32]. Connection between *mUzima* and the EHR system server occurs using a representational state transfer (REST) API [33].

The *mUzima* API includes the business logic layer of the platform, exposing the underlying Lucene repository to a robust front-end application (*mUzima* Android) while hiding the complexity of the underlying repository to the developer. This layer can be easily used to expose the *mUzima* data model to any system integrating with *mUzima*.

*mUzima* Android is the Android application package (APK) with which users interact, oblivious of the other 2 underlying stack layers. *mUzima* Android supports user interface activities and uses the underlying *mUzima* API to store and retrieve data resources from the phone’s local repository and to upload and download data from the remote EHR system server. The APK is structured into the following main components: (1) models comprised of objects that encapsulate data and logic for resources specific to Android. Some of the objects extend *mUzima* API models and add logic specific to usage in the Android application and (2) controllers for each type of resource. The controllers act as coordinators within the Android app and between the Android app and *mUzima* API. Controllers are commonly used to request various actions related to objects, such as sending to storage, retrieval from storage, searching, counting, uploading, and downloading; (3) services, which are components used to handle requests for repetitive and potentially long-running operations; and (4) views, which are the implementation of the *mUzima* user interface.

#### *mUzima* Server-Side Module

**Figure 3:**
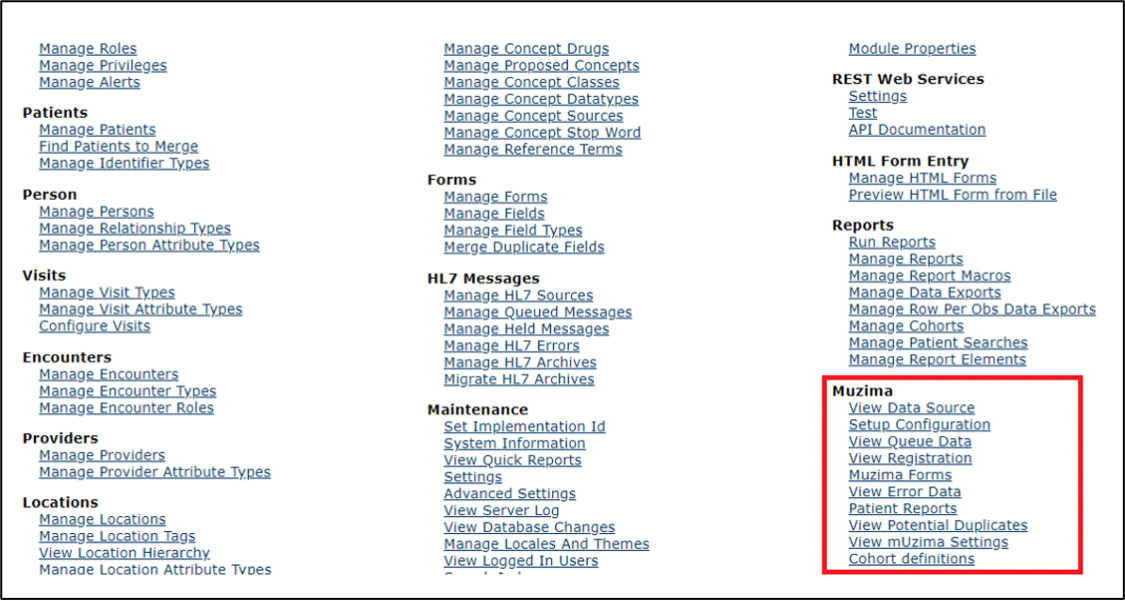
Components of the *mUzima* server-side module.

*mUzima* has a server-side module created within the OpenMRS EHR system that has REST endpoints enabling linkage of OpenMRS instances to the *mUzima* mobile application (Figure 3 and Multimedia Appendix 1). Key features of the *mUzima* server-side module include (1) the set-up configuration and (2) error resolution mechanisms. The server-side setup configuration features are used to define care programs. To this end, the setup configuration has provisions for defining patient lists or cohorts, forms, historical data, providers, and locations to be included in each care program (Multimedia Appendix 1). The server side also offers the ability to define multiple settings that will be applied onto all *mUzima* instances on mobile devices, removing the labor-intensive need of having to configure individual settings within each mobile device. The error resolution mechanism on the server side allows nondevelopers to manage forms submitted to the server that are identified as having errors (Multimedia Appendix 2). Errors are typically encountered when information on clients registered offline are synchronized onto the server and are found to be duplicates once patient-matching is run on the server. The ability to resolve errors on submitted data without the need for programming expertise or access to the server’s backend makes *mUzima* highly usable and scalable in remote facilities, which often lack personnel to offer advanced technical support. The error resolution module also handles queuing and processing of data submitted into OpenMRS from *mUzima.*

#### *mUzima* Mobile Application

**Figure 4:**
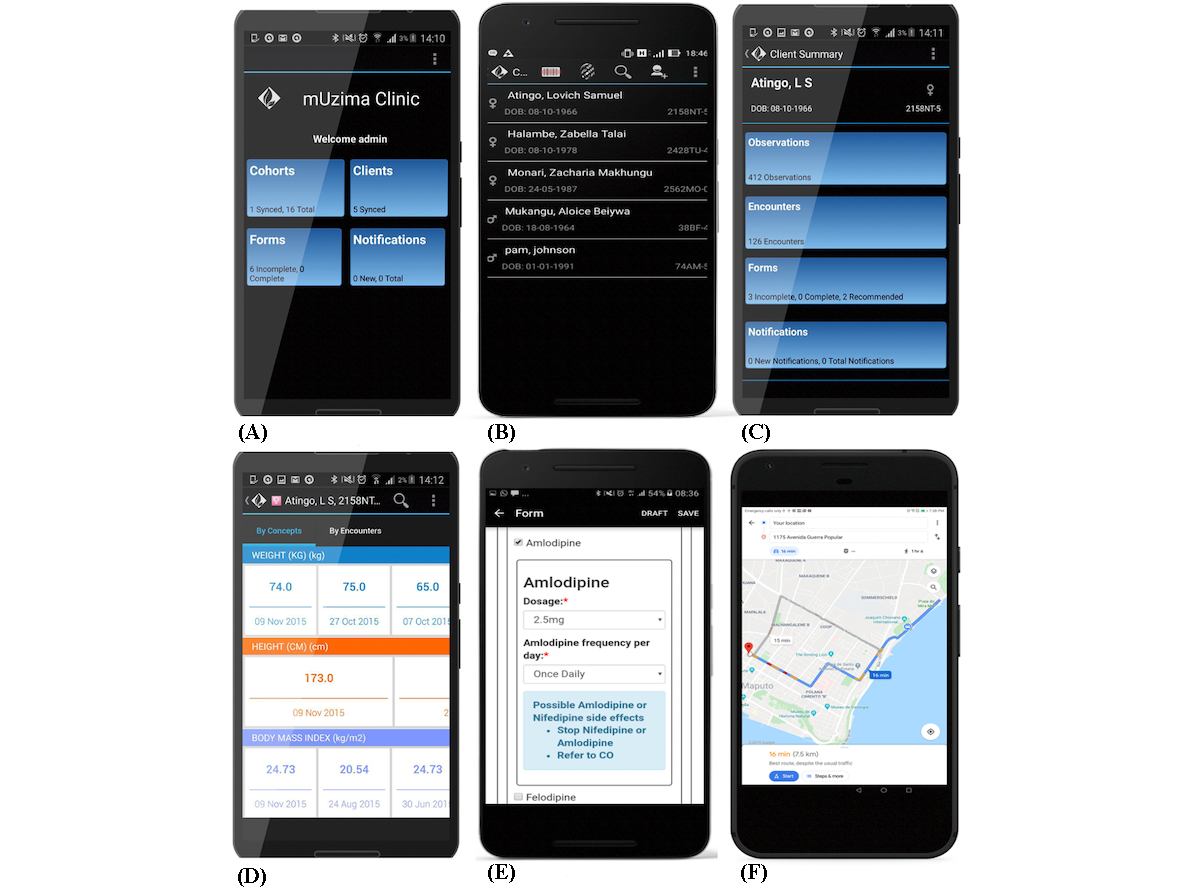
*mUzima* features: (A) menu options, (B) client list, (C) options under client, (D) historical data, (E) form entry with decision support, (F) geolocation.

Access to the *mUzima* mobile application requires a username and password, which are initially authenticated against the EHR system. This access times out automatically if the user is idle for a predefined period of time. Once logged in, users have access to the *mUzima* mHealth application landing page, which can be defaulted to display key menu options of cohorts, forms, clients, and notifications (Figure 4A). Alternatively, the landing page can be configured to display lists of clients available on the device (Figure 4B). *mUzima* users are able to find clients by scrolling through the client list, by searching via names and IDs, or by using the barcode scanning feature. Partial matching of names is available against both the local Lucene-based database that provides fast search capabilities [28] and the linked OpenMRS instance via its RESTful API. Further details of specific *mUzima* features are outlined in the Item 4 and Item 5 sections that follow.

### Item 3: Interoperability and Health Information System (HIS) Context

*mUzima* serves as an HIS for collecting primary health data, which are then exchanged with the associated EHR system. Health information exchange between *mUzima* and OpenMRS is achieved through use of the same concept dictionary terms, locations, providers, and patient identifiers that are common between the 2 systems. The REST API is used to connect *mUzima* to OpenMRS for bidirectional data exchange (Figure 1) [33]. This multifaceted approach for direct system-to-system exchange has been adopted to overcome the lagging implementation of robust health information exchange mediators and mechanisms in many LMICs. To facilitate future integration and health information exchange with other systems, several fast health care interoperability resources (FHIR) such as patient, provider, and observation FHIR are being developed for *mUzima* [34].

### Item 4: Intervention Delivery

The WHO, in its “Recommendations on digital interventions for health system strengthening,” strongly advocates that “recommended interventions should be accessible via mobile devices at a minimum” [35]. *mUzima* is primarily for use by health providers through smartphones that are often owned by the care program. Access to *mUzima* is on-demand when the provider logs in with the relevant credentials. The providers use *mUzima* to access cohorts and historical data on patients under their care, as well as to capture new data on patients. Significant time and effort were expended to ensure that *mUzima* can auto-update changes in cohort membership, forms, and historical data elements (ie, managing delta), without the need to re-download data that had previously been downloaded onto the mobile device. This allows for seamless update of content to the mobile device through *mUzima’s* synchronization mechanism with OpenMRS.

### Item 5: Intervention Content

Over half of the recommended digital interventions by the WHO pertain to mobile-based EHR solutions and include “digital tracking of clients’ health status combined with decision support; targeted client communication; digital provision of educational and training content to health workers; provider-to-provider telemedicine; and client-to-provider telemedicine” [35]. *mUzima* satisfies all of the WHO mobile-based EHR system recommendations. Core features currently available within the *mUzima* application are described in the following sections.

#### Cohort Management

Given limitations on storage size within mobile devices, approaches are needed that limit the amount of data downloaded onto devices. This means downloading only the relevant subset of patients and their corresponding data to the mobile device. *mUzima* features the ability to define cohorts of patients using SQL. Cohorts can be as simple as a list of individuals with a particular disease (eg, diabetes) or can be more complicated, such as “diabetic patients with kidney disease plus poorly controlled blood sugar and who are noncompliant with their medications.” *mUzima* has functionality that allows for membership within a cohort to remain “static” (ie, never change after the initial run) or to be dynamic (allowing for automatic addition or removal of members over time, based on whether they still meet the cohort criteria).

#### Programs

A program within *mUzima* is used to consolidate all features in the mHealth application that are needed to take care of a group of patients. Programs can be disease-specific or based on cohorts. *mUzima* provides the ability to configure all details for programs using the set-up configuration feature on the server side (see the *mUzima* Server-Side Module section). Programs contain (1) lists of clients from one or more cohorts, (2) details of relevant historical data elements to be downloaded onto the device for these clients, (3) program-specific forms to be completed for clients, (4) an optional list of locations and providers that serve the program, and (5) settings that can be predefined for end users. Program functionality enables *mUzima* to be highly configurable and disease-agnostic, allowing the application to serve multiple clinical scenarios and domains. Further, programs integrate workflow features, such as sequential filling of forms, to support care processes. The ability to easily configure elements for programs ensures needed optimization of mobile device setup and also reduces the cost and time of data transmission and downloads.

#### Historical Data

Through its connection with an EHR system, *mUzima* has the ability to download a subset of historical data on patients that are predefined as part of a program. Beyond program-defined historical data to be downloaded, users of the *mUzima* application have the option to include additional historical data or remove any or all of the predefined historical data, based on their individual preferences. The historical data downloaded through *mUzima* can come from any source that saves data to the EHR system, including laboratory and pharmacy systems, ensuring that providers using *mUzima* have the complete clinical picture of a patient’s data. This contrasts with numerous mHealth applications that only display historical data originally collected through that application and not from any other sources or those that are used as simple data collection tools, with no ability to display any historical information. Within *mUzima,* the historical data can be viewed by data type, date, or clinical encounter type (Figure 4D).

#### Data Capture Mechanisms

*mUzima* allows flexibility in data collection, with forms created to capture elements based on the same concept dictionary terms and other patient attributes (eg, demographics) shared with the EHR system. HTML5 is used to describe each form, enabling form development with little programming experience. *mUzima* forms can accommodate all data types, such as text, numeric, float, Boolean, and dates, and can support numerous media types. The application can also capture individual data elements (eg, individual test results), without relying on a form. As needed, *mUzima* forms can incorporate data validation, branching logic, rule-based decision support, and multimedia items (Figure 4E). Data captured within forms can be saved as complete or as draft for future completion. A configurable autosave feature also exists to ensure that data collected in forms are not inadvertently lost.

Forms are automatically downloaded into the mobile application based on their inclusion within a program, but mobile device users can easily add or remove individual forms as per the user’s preferences. With *mUzima,* there is no need to install a new version of the application every time new forms or form updates are available. Further, the *mUzima* form update mechanism is optimized to ensure that no data collected in older forms within the mobile application are lost during the form update process.

#### Registration and Patient Matching

Registration forms within *mUzima* rely on the form features. Prior to registering any new client, the application runs a probabilistic patient-matching algorithm to ensure that duplicate patients are not created. When no patient matches are found on the mobile device, *mUzima* runs a match against the server if there is connectivity. In settings where the device is offline and new individuals still need to be registered offline, the *mUzima* server-side module provides functionality to first match the new registration against existing EHR records once the registration information is uploaded to the server. If duplicates are found, functionality exists to merge the patient’s records.

#### Geolocation Services

Patient tracing is a core component of community-based care, especially in areas without reliable address systems. Geolocation functionality within *mUzima* can help in finding patients within communities (Figure 4G). *mUzima* provides the ability to add and edit the GPS locations for individuals and employs navigation functionality to help locate patients. Functionality also exists to automatically capture the location where a clinical form is completed, should the program decide to enable this feature.

#### Relationship Support

The relationships feature captures the nature of relationships of various individuals with the reference client. Relationships are particularly relevant for contact tracing in the age of COVID-19 and for HIV index case testing services. In both cases, individuals related to or who have contact with the index case need to be traced for testing and follow-up. *mUzima* supports capturing information on the nature of the relationship between 2 individuals and collection of data on the related individual (Multimedia Appendix 3).

Table 1 summarizes key features within the *mUzima* application, highlighting often-overlooked elements in developing configurable and scalable mHealth applications for use in LMICs [36].

**Table 1:** *mUzima* features.

Feature	Description
EHR^a^ system compatibility	*mUzima* was designed to be the mHealth^b^ extension to a largely deployed EHR system (OpenMRS) to prevent siloed data collected within *mUzima*. OpenMRS is deployed nationally in several countries.
Security	Security features in *mUzima* include password-based log-in, data encryption, secure data transmission, timed user logouts, and a password-changing mechanism within limited connectivity settings.
Multiple use cases	*mUzima* is easily customizable to support any clinical use case, both within and outside of clinical facilities.
Data collection tools	*mUzima* uses easy-to-develop, web-based forms to collect data (including by providers off-site) that are securely transmitted and stored in an EHR system, as opposed to a siloed server
Offline capabilities	*mUzima* functions smoothly in both online and offline modes. This allows for data collection and review, even when the mobile device is offline.
Error resolution	*mUzima* prevents duplicate form data entry on mobile devices and resolves errors in the EHR system during data processing.
Form management	*mUzima* enables easy download and update of new forms onto the app without the need to re-install or restart the app. Form default text sizes are configurable, with the ability to magnify forms during use.
Cohort management	*mUzima* allows for the management of different patient or client groups on a mobile device.
Multiple languages	*mUzima* provides localization support for use in various languages. The full application is also currently available in 6 languages: English, Portuguese, French, Swahili, Gujarati, and Hindi.
Multiple themes	*mUzima* is shipped on 2 themes as of v2.5.0, including a dark theme (white on black) and light theme (black on white)
Relationships	In the health care space, a relationship is used to pair or associate 2 people whose care may be interlinked. *mUzima* supports recording of relationships (Multimedia Appendix 3).
Geomapping	*mUzima* can easily capture and record patient GPS coordinates and provides navigation services to support tracing of clients (Multimedia Appendix 4).
Clinical summary or abstracts	*mUzima* utilizes the OpenMRS Reporting Module to design and generate HTML reports that are then rendered on the app, providing comprehensive abstracts for each patient (Multimedia Appendix 4).

^a^EHR: electronic health record.

^b^mHealth: mobile health.

### Items 6: Usability and Content Testing

In line with the Digital Development Principles of “Building for Sustainability” and “Be Collaborative,” *mUzima* relies on a community of developers and implementers for content and user acceptance testing. As of February 2021, the *mUzima* community had 112 members, with a total of 31 unique contributors to the *mUzima* codebase. This community has made over 3800 contributions or commits to the codebase. Over 250 individuals have used, contributed, or reviewed *mUzima* documentation through *mUzima*’s Wiki platform [37], and more than 190 have contributed in the *mUzima* discussion forums [38]. The community is made up of stakeholders supported through various funded projects and agencies.

User testing for *mUzima* is conducted by a core team, as well as by community members prior to any *mUzima* release. Each feature in an *mUzima* release is documented as a JIRA ticket [39,40]. These tickets are used to track testing findings, with changes made based on feedback received. Final acceptance testing is conducted prior to deploying a new version of the application. Multimedia Appendix 5 provides a screenshot of release testing for a ticket in *mUzima* version 2.7.0, with details of testing status for each ticket in this version shared in a publicly available release plan [40].

### Item 7: User Feedback

A user-centered design approach is employed in developing core *mUzima* functionality and features and in adapting the application for various use cases. An engaged Implementer Community provides suggestions for features, as well as feedback on developed application components and content. This feedback is primarily gathered via weekly community calls and through the *mUzima* forum [38]. Additional user inputs are gathered through reviews of the application using the feedback mechanism in Google Play Store, where *mUzima* has thousands of downloads and a 5-star rating [41].

### Item 8: Access by Individual Participants

*mUzima* increases access to clinical data for providers who work outside of care facilities and in settings disconnected from the EHR system server. It is a provider-facing application and not available for direct use by patients. The application can leverage both WiFi-based or phone-based data bundles for data transmission. As an Android application, *mUzima* is not available to health providers who do not have Android smartphones and has limited utility to providers who are illiterate or unfamiliar with using smartphones. To use *mUzima,* providers need to have credentials to access an OpenMRS EHR instance, and this will limit utility of *mUzima* for those without such access or those using other EHR systems.

### Item 9: Cost Assessment

A formal cost assessment has yet to be done on *mUzima.* Although *mUzima* is available free of charge to users, there are still several associated costs to implement it. Key costs include customization costs that involve creating relevant forms for a program; development of queries that define cohorts of patients; smartphone purchase; costs associated with hosting an OpenMRS instance (if one does not exist); connectivity costs to the server through WiFi or Internet; and costs of personnel to support users, maintain infrastructure, and conduct training. Comparatively, in instances where care is conducted outside of facilities, *mUzima* implementation should be cheaper than using laptops equipped with OpenMRS—given the higher cost associated with laptops and managing multiple OpenMRS instances. Although paper-based systems have historically been used to support care in LMICs, they have recurrent costs given that pieces of paper can only be used once to record data, and there are personnel costs associated with retrospective entry of data collected on paper into the EHR system [42]

### Item 10: Adoption Inputs and Program Entry

*mUzima* is freely available on the Google Play Store [41], with all changes and enhancements in any new version shared broadly via multiple channels, as well as fully documented in the collaborative wiki space [37]. Within the wiki space are documents on setting up the system, as well as training materials. Multiple introductory and educational videos on *mUzima* are also available through the *mUzima* YouTube channel [43]. *mUzima* training often involves technical personnel as well as training of end user providers. Technical team members, comprised of developers and implementing information technology personnel, often need to understand how to customize *mUzima* and to set up the server and smartphone devices for use. The technical team training is largely hands-on, with the *mUzima* community forums and weekly meetings available for answering questions from community members. End users are often trained by the technical team members from their organization. These trainings typically cover core *mUzima* features and also the customized elements relevant to that program. For most end users, training on *mUzima* typically takes 1 to 2 days. Refresher trainings are available especially when there are upgrades to the application.

### Item 11: Limitations for Delivery at Scale

The Digital Development Principle “Design for scale” is highly relevant to *mUzima.* Scalability was considered at the outset, allowing for the system to support any type of clinical condition, various workflows, and multiple versions of both OpenMRS and Android. Load testing is a core part of all *mUzima* version releases to ensure that *mUzima* works with larger patient populations and data needs. *mUzima’s* ability to scale is evidenced by its successful large-scale adoption across multiple clinical settings and countries (Table 2). As a testament of its scalability and implementation success, *mUzima* has been recognized as a digital global public good since 2019 [22] and is listed as a global project in the WHO “Digital Health Atlas” [44].

Despite having scaled to national levels, further expansion of *mUzima* is limited by the fact that it only currently extends 1 EHR system, OpenMRS. *mUzima’s* FHIR resources are under development to allow seamless health information exchange with other FHIR-compliant systems [34]. Customization of *mUzima* requires knowledge of form programming using HTML and basic knowledge on creating cohorts and queries within OpenMRS—both of which can limit scaling of *mUzima* implementations. Further, as a native Android application, *mUzima* will not work with smartphones that run on different operating systems.

**Table 2:** Large-scale *mUzima* implementations.

Type of implementation	Description
HIV care	*mUzima* is nationally endorsed and implemented by the Kenya Ministry of Health (MoH) to support HIV testing and screening (HTS) [45]. In Kenya, the application is in use at over 220 public facilities, with more than 1000 providers using it on any given workday. To date, over 500,000 HTS visits have been conducted in Kenya using *mUzima*. In addition, *mUzima* is in use in Zambezia province in Mozambique for defaulter and lost-to-follow-up tracing and for preventative visits (Multimedia Appendix 6). The application has also been adapted for national rollout in support of HIV patient tracing activities in Uganda.
Cancer care	Partners in Health Rwanda, working closely with the Rwanda MoH and the Clinton Health Access Initiative, are using *mUzima* for cervical and breast cancer screening and referral. Currently implemented in 3 districts, the *mUzima* application has already been used to support data collection during screening of more than 4000 patients for breast and cervical cancer at 39 facilities. The goal is to extend *mUzima* application use to 15 districts in Rwanda.
Chronic disease management (CDM)	*mUzima* is in use at more than 70 facilities in Kenya as part of a task-shifting program to support CDM, including hypertension and diabetes care. Unlike other implementations that use *mUzima* in communities, these CDM implementations use *mUzima* at dispensaries and within other facilities across 6 counties (Uasin Gishu, Bungoma, Trans-Nzoia, Nandi, Kisumu, and Busia). To date, over 350 providers have used *mUzima* to record CDM encounters in more than 100,000 visits. The CDM program incorporates robust decision support features based on alerts and reminders, as well as educational media with a personalized display based on patient-specific data (Figure 4E).
COVID-19	At the request of Kenya’s MoH, *mUzima* was adapted to support COVID-19 care, including screening, contact tracing, testing, and symptom monitoring. The COVID-19 forms used are based on guidelines provided by the World Health Organization (WHO) and use standard terms based on the Columbia International eHealth Laboratory (CIEL) dictionary [46]. The *mUzima* COVID-19 application leverages relationship and geomapping features for contact tracing and is available for adaptation beyond Kenya (Multimedia Appendix 7). *mUzima* is also in use by the MoH in Rwanda for COVID-19 management.

### Item 12: Contextual Adaptability

*mUzima* has been used across various health domains (Table 2), with most use in primary contexts that have limited internet connectivity, especially in Sub-Saharan Africa. *mUzima* provides localization support for use in various languages and is currently available in 6 languages, namely English, Portuguese, French, Swahili, Gujarati, and Hindi. The application comes with multiple configurable settings that are not hard coded into the application. Examples of configurable settings include whether to capture GPS location of activity, default font size, auto-synchronization feature, time setting for automatic time-outs, form auto-save time interval, among other features. Forms, cohorts, and clinical observations within the application can be customized without any need for reprogramming.

### Item 13: Replicability

Content related to *mUzima* is organized in the application’s website that highlights key application features and provides a demonstration instance for those interested in exploring the application [21]. This website provides links to all other *mUzima-*related resources and documentation. Detailed documentation on *mUzima* is available on the wiki page and includes user guides, technical documentation, and implementation guides, among others [37]. Multimedia content such as demonstration videos is also available on the *mUzima* YouTube channel [43]. *mUzima* code is shared via GitHub [47], and as of February 2021, there had been 650 pull requests and over 120 forks to the *mUzima* code.

### Item 14: Data Security

*mUzima* is developed to enable the primary clinical implementing partners to remain full custodians of all data collected and transmitted to OpenMRS through *mUzima*. As such, identifiable patient information is only accessible to those given access through the EHR system. In line with the Principle for Digital Development to “Address privacy and security,” the *mUzima* application has incorporated several security features. Access to the *mUzima* is done through a username and password validated initially with the EHR system. Users who forget their password have to be re-authenticated within the EHR system by an administrator. *mUzima* contains a feature for automatic time-outs where users are logged out after a program-defined period of inactivity. Bidirectional exchange of data between *mUzima* and the EHR system is secured through use of an https protocol. All *mUzima*-related data are stored in an encrypted format in the mobile device.

### Item 15: Compliance With National Guidelines or Regulatory Statutes

Implementations using *mUzima* have full control of locations of servers where patient data from *mUzima* are stored. It is advocated that these implementations comply with data residency guidelines and laws for the country of operation. Organizations that opt to use *mUzima* are advised to contact relevant statutory bodies within countries that oversee mHealth applications. *mUzima* allows users to configure forms, patient identifiers, and concept dictionary terms to be used in each implementation. Compliance with national guidelines for these items is thus at the discretion of the implementers but is highly advocated. Forms and data elements can be easily developed to align with approved forms in countries and using any national dictionary should one be available. As an example, implementations in Kenya leverage the standards-based Columbia International eHealth Laboratory (CIEL) concept dictionary [46].

### Item 16: Fidelity of the Intervention

Performance of the *mUzima* application is gathered using the Firebase Crashlystics plugin [48]. These performance metrics indicate that it takes <5 seconds for the application to launch and <1 second to upload each completed form payload to the OpenMRS EHR system from within *mUzima*. Over the most recent 3-month period (December 2020 to February 2021), the percentage of *mUzima* users without crashes in any of their usage sessions was 92.88%. *mUzima* also uses Firebase Crashlystics to monitor locations where *mUzima* has been used over a period of time, as well as high-level details of the users, broken down by user-provided gender and age (Multimedia Appendix 8). For individualized and detailed usage statistics, *mUzima* has also been programmed to collect paradata through logs of key components of the application while it is in use. Although performance and workflow analytics can be conducted on the logged paradata, there is a need to create a user-friendly and personalized dashboard highlighting a user’s work performance on *mUzima* that can be availed to support decision making around work patterns.

## Discussion

Although EHR and mHealth solutions have been widely embraced in LMICs, there is often lack of seamless data exchange between widely deployed EHR systems and these mHealth solutions. To our knowledge, this is one of the only descriptions of a robust extension of a widely used EHR system in LMICs. Most other widely deployed mHealth applications primarily save data to their own servers and have challenges integrating with existing EHR systems. It will be several years before all LMICs have effective interoperability and health information exchange infrastructure [49,50]. In the meantime, a paradigm that advocates for comprehensive mobile extensions to existing EHR systems will help to reduce the widening digital divide that adversely impacts resource-limited settings.

For mHealth applications to be effective and scalable within resource-limited settings, they have to build on lessons learned in implementation and development over the years [16,19,35]. Integration of mHealth applications with EHR systems uncovers new challenges that deserve attention. In particular, challenges emerge around best approaches to manage user credentials across 2 systems and in disconnected settings, appropriate delivery of subsets of EHR system data to mobile devices that have limited storage, management of ever-changing cohort sizes and patient-level data while ensuring data transmission costs are contained, and development of tethered mHealth solutions that can adapt to multiple use cases without the need to reprogram the application.

The *mUzima* team is currently broadening its support for implementations within various countries to ensure responsiveness to needs. As countries mature with regard to health information exchange, the *mUzima* team has started implementing FHIR, starting with patient, person, encounter, and observation FHIR [34]. The application user interface is undergoing redesign and revamping with input from users. The aim is primarily to enhance the user experience and to ensure that key features are more easily accessible to users through the interface. The *mUzima* team is in the process of adding secure messaging and communication features and incorporating a more robust teleconsultation feature, beyond the store-and-forward teleconsultation functionality already incorporated in the application. Finally, the application’s modularization feature is being revamped to enable easier incorporation of additional plug-ins to *mUzima*. The *mUzima* data analytics team is also working on reusable paradata analytics and visualization to provide needed information on provider work performance and engagement with the application [51].

Evaluation is important for any health information system. Usability and feasibility of *mUzima* have previously been assessed in an implementation to support nurses in managing hypertensive patients [52]. Ongoing *mUzima* evaluations include (1) evaluation of patient-specific, phone-generated reminders through *mUzima* for hypertension care in Kenya; (2) evaluating use of *mUzima*-facilitated monitoring of health worker performance in Western Kenya using logged paradata; and (3) evaluation of the *mUzima* mobile application for patient tracing and preventive visits in Mozambique. Priorities for future evaluations should include comprehensive assessment of costs and benefits of using the *mUzima* application.

In conclusion, greater emphasis needs to be placed on mHealth applications that extend reach of EHR systems within resource-limited settings to reduce the digital divide that has emerged with use of standalone EHR systems or mHealth applications. *mUzima* demonstrates how this can be done at scale, with evident adoption across countries and for various types of care programs.

## Appendix

Multimedia Appendix 1 <italic>mUzima</italic> Server-Side Setup Configuration.

Multimedia Appendix 2 <italic>mUzima</italic> Error Resolution Mechanism.

Multimedia Appendix 3 <italic>mUzima</italic> Relationship Features.

Multimedia Appendix 4 <italic>mUzima</italic> Geo-mapping feature & Clinical Summary.

Multimedia Appendix 5 Screenshot of Release Testing for a Ticket in <italic>mUzima</italic> Version 2.7.0.

Multimedia Appendix 6 <italic>mUzima</italic> for Patient Tracing in Mozambique.

Multimedia Appendix 7 <italic>mUzima</italic> for COVID-19.

Multimedia Appendix 8 Locations of <italic>mUzima</italic> App Usage.

## Abbreviations

API: application programming interface
APK: Android application package
CIEL: The Columbia International eHealth Laboratory
EHR: electronic health record systems
FHIR: fast health care interoperability resources
HIS: health information system
LMICs: low- and middle-income countries
mERA: mHealth Evidence Reporting and Assessment
mHealth: mobile health
MoH: Ministry of Health
NORHED: Norwegian Programme for Capacity Development in Higher Education and Research for Development
REST: representational state transfer
USAID: United States Agency for International Development
WHO: World Health Organization

## References

[1] Williams F, Boren S (2008). The role of the electronic medical record (EMR) in care delivery development in developing countries: a systematic review. Inform Prim Care.

[2] Syzdykova A, Malta A, Zolfo M, Diro E, Oliveira JL (2017). Open-source electronic health record systems for low-resource settings: systematic review. JMIR Med Inform.

[3] Lewis T, Synowiec C, Lagomarsino G, Schweitzer J (2012). E-health in low- and middle-income countries: findings from the Center for Health Market Innovations. Bull World Health Org.

[4] Muinga N, Magare S, Monda J, Kamau O, Houston S, Fraser H, Powell J, English M, Paton C (2018). Implementing an open source electronic health record system in Kenyan health care facilities: case study. JMIR Med Inform.

[5] (2020). Implementation of the UgandaEMR: Results of a Security Assessment. MEASURE Evaluation.

[6] Chaplin B, Meloni S, Eisen G, Jolayemi T, Banigbe B, Adeola J, Wen C, Reyes Nieva H, Chang C, Okonkwo P, Kanki P (2015). Scale-up of networked HIV treatment in Nigeria: creation of an integrated electronic medical records system. Int J Med Inform.

[7] (2016). eSaúde: Building a Local OpenMRS Community to Support a National EMR Implementation in Mozambique. OpenMRS.

[8] Nsanzimana S, Kanters S, Remera E, Forrest JI, Binagwaho A, Condo J, Mills EJ (2015). HIV care continuum in Rwanda: a cross-sectional analysis of the national programme. The Lancet HIV.

[9] (2014). Fast Track: ending the AIDS epidemic by 2030. UNAIDS.

[10] Levi J, Raymond A, Pozniak A, Vernazza P, Kohler P, Hill A (2016). Can the UNAIDS 90-90-90 target be achieved? A systematic analysis of national HIV treatment cascades. BMJ Glob Health.

[11] Kang'a S, Puttkammer N, Wanyee S, Kimanga D, Madrano J, Muthee V, Odawo P, Sharma A, Oluoch T, Robinson K, Kwach J, Lober WB (2017). A national standards-based assessment on functionality of electronic medical records systems used in Kenyan public-Sector health facilities. Int J Med Inform.

[12] Tweya H, Feldacker C, Gadabu OJ, Ng'ambi W, Mumba SL, Phiri D, Kamvazina L, Mwakilama S, Kanyerere H, Keiser O, Mwafilaso J, Kamba C, Egger M, Jahn A, Simwaka B, Phiri S (2016). Developing a point-of-care electronic medical record system for TB/HIV co-infected patients: experiences from Lighthouse Trust, Lilongwe, Malawi. BMC Res Notes.

[13] Were MC, Emenyonu N, Achieng M, Shen C, Ssali J, Masaba JPM, Tierney WM (2010). Evaluating a scalable model for implementing electronic health records in resource-limited settings. J Am Med Inform Assoc.

[14] Muthee V, Bochner A, Kang'a S, Owiso G, Akhwale W, Wanyee S, Puttkammer N (2018). Site readiness assessment preceding the implementation of a HIV care and treatment electronic medical record system in Kenya. Int J Med Inform.

[15] Were M, Sinha C, Catalani C (2019). A systematic approach to equity assessment for digital health interventions: case example of mobile personal health records. J Am Med Inform Assoc.

[16] Lopéz DM, Blobel B (2015). mHealth in low- and middle-income countries: status, requirements and strategies. Stud Health Technol Inform.

[17] Garner SL, Sudia T, Rachaprolu S (2018). Smart phone accessibility and mHealth use in a limited resource setting. Int J Nurs Pract.

[18] Kruse C, Betancourt J, Ortiz S, Valdes Luna SM, Bamrah IK, Segovia N (2019). Barriers to the use of mobile health in improving health outcomes in developing countries: systematic review. J Med Internet Res.

[19] Labrique AB, Wadhwani C, Williams KA, Lamptey P, Hesp C, Luk R, Aerts A (2018). Best practices in scaling digital health in low and middle income countries. Global Health.

[20] Health Information Systems Interoperability Maturity Toolkit. MEASURE Evaluation.

[21] mUzima.

[22] Digital Public Goods Definition. Digital Public Goods Alliance.

[23] Agarwal S, LeFevre AE, Lee J, L'Engle K, Mehl G, Sinha C, Labrique A, WHO mHealth Technical Evidence Review Group (2016). Guidelines for reporting of health interventions using mobile phones: mobile health (mHealth) evidence reporting and assessment (mERA) checklist. BMJ.

[24] Principles for Digital Development.

[25] OpenMRS.

[26] Mamlin BW, Biondich PG, Wolfe BA, Fraser H, Jazayeri D, Allen C, Miranda J, Tierney WM (2006). Cooking up an open source EMR for developing countries: OpenMRS - a recipe for successful collaboration. AMIA Annu Symp Proc.

[27] OpenMRS Atlas. OpenMRS.

[28] Mobile Operating System Market Share Africa. Statcounter.

[29] Mozilla Public License Version 2.0. Mozilla Foundation.

[30] Apache Lucene.

[31] google / guice. GitHub.

[32] Indexer (programming). Wikipedia.

[33] (2017). RESTful Services - HTTP. Sagar Mane.

[34] Mandel J, Kreda DA, Mandl KD, Kohane IS, Ramoni RB (2016). SMART on FHIR: a standards-based, interoperable apps platform for electronic health records. J Am Med Inform Assoc.

[35] (2019). WHO Guideline: recommendations on digital interventions for health system strengthening. World Health Organization.

[36] Features. mUzima.

[37] muzima. mUzima Wiki.

[38] mUzima Forums. mUzima.

[39] JIRA Software. Atlassian.

[40] (2020). mUzima 2.7.0 Release plan. mUzima Wiki.

[41] mUzima. Google Play Store.

[42] Were M, Kariuki J, Chepng'eno V, Wandabwa M, Ndege S, Braitstein P, Wachira J, Kimaiyo S, Mamlin B (2010). Leapfrogging paper-based records using handheld technology: experience from Western Kenya. Stud Health Technol Inform.

[43] (2020). Introducing mUzima for COVID-19. youtube.

[44] mUzima. Digital Health Atlas.

[45] Farmer B (2018). What The U.S. Could Learn From Vanderbilt’s Electronic Health Records Experiment In Kenya. WPLN News.

[46] Blaya J, Mamlin B (2020). The MVP-CIEL Concept Dictionary. OpenMRS Wiki.

[47] mUzima. GitHub.

[48] Firebase Crashlytics. Firebase.

[49] Kumar M, Mostafa J (2019). Research evidence on strategies enabling integration of electronic health records in the health care systems of low- and middle-income countries: A literature review. Int J Health Plann Manage.

[50] Higman S, Dwivedi V, Nsaghurwe A, Busiga M, Sotter Rulagirwa H, Smith D, Wright C, Nyinondi S, Nyella E (2019). Designing interoperable health information systems using Enterprise Architecture approach in resource-limited countries: A literature review. Int J Health Plann Manage.

[51] Hightow-Weidman LB, Bauermeister JA (2020). Engagement in mHealth behavioral interventions for HIV prevention and care: making sense of the metrics. Mhealth.

[52] Vedanthan R, Blank E, Tuikong N, Kamano J, Misoi L, Tulienge D, Hutchinson C, Ascheim DD, Kimaiyo S, Fuster V, Were MC (2015). Usability and feasibility of a tablet-based Decision-Support and Integrated Record-keeping (DESIRE) tool in the nurse management of hypertension in rural western Kenya. Int J Med Inform.

