# Supplementary figures and images for: mUzima Mobile Electronic Health Record (EHR) System: Development and Implementation at Scale
Source: J Med Internet Res. 2021 Dec 14;23(12):e26381. doi: 10.2196/26381 (PMC8715359; doi:10.2196/26381)

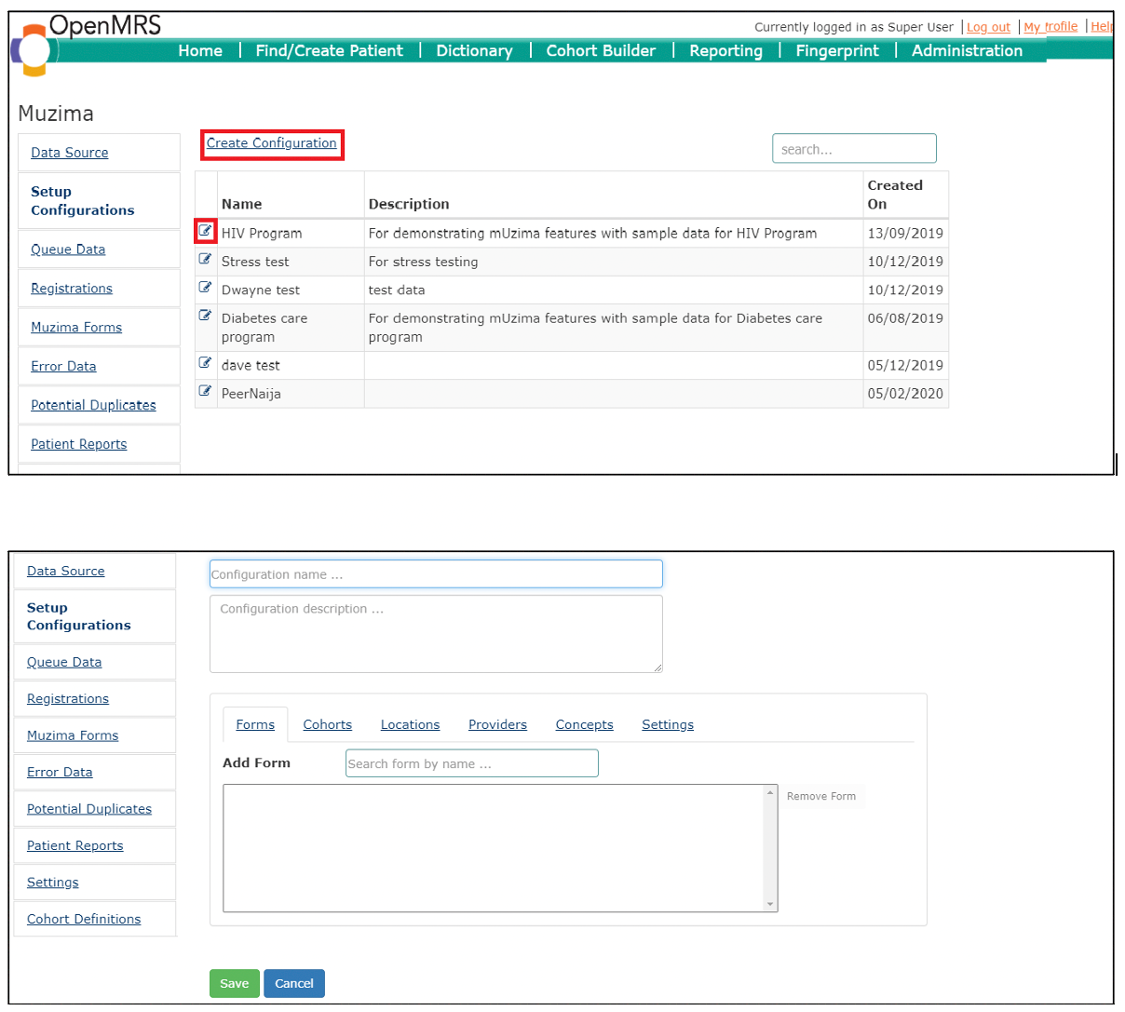

Supplement: Multimedia Appendix 1 [file jmir_v23i12e26381_app1.png]

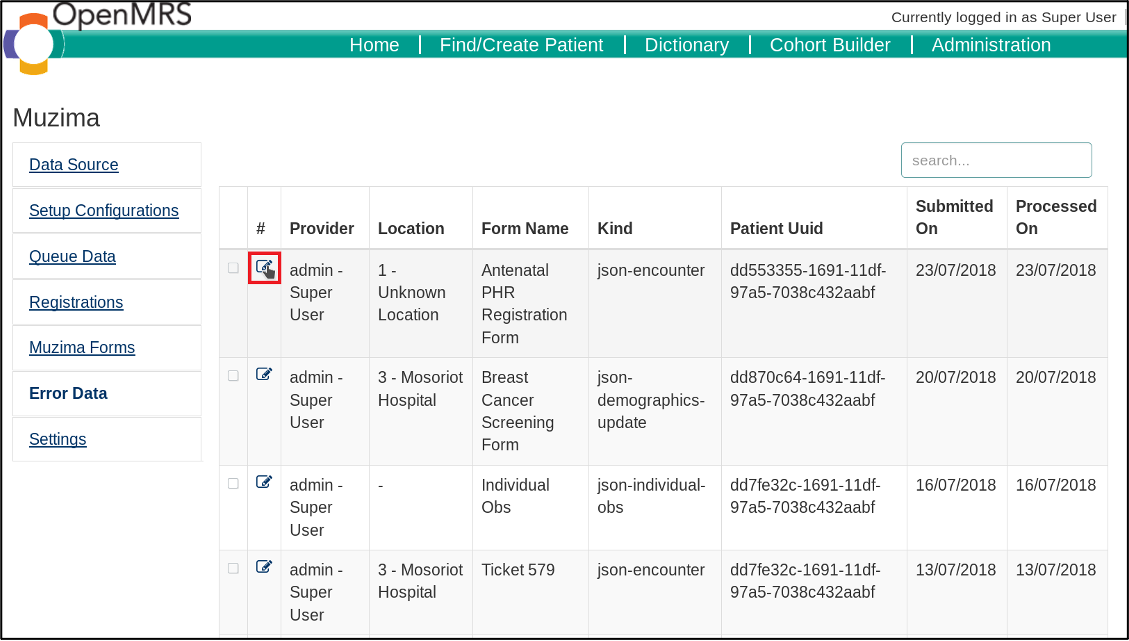

Supplement: Multimedia Appendix 2 [file jmir_v23i12e26381_app2.png]

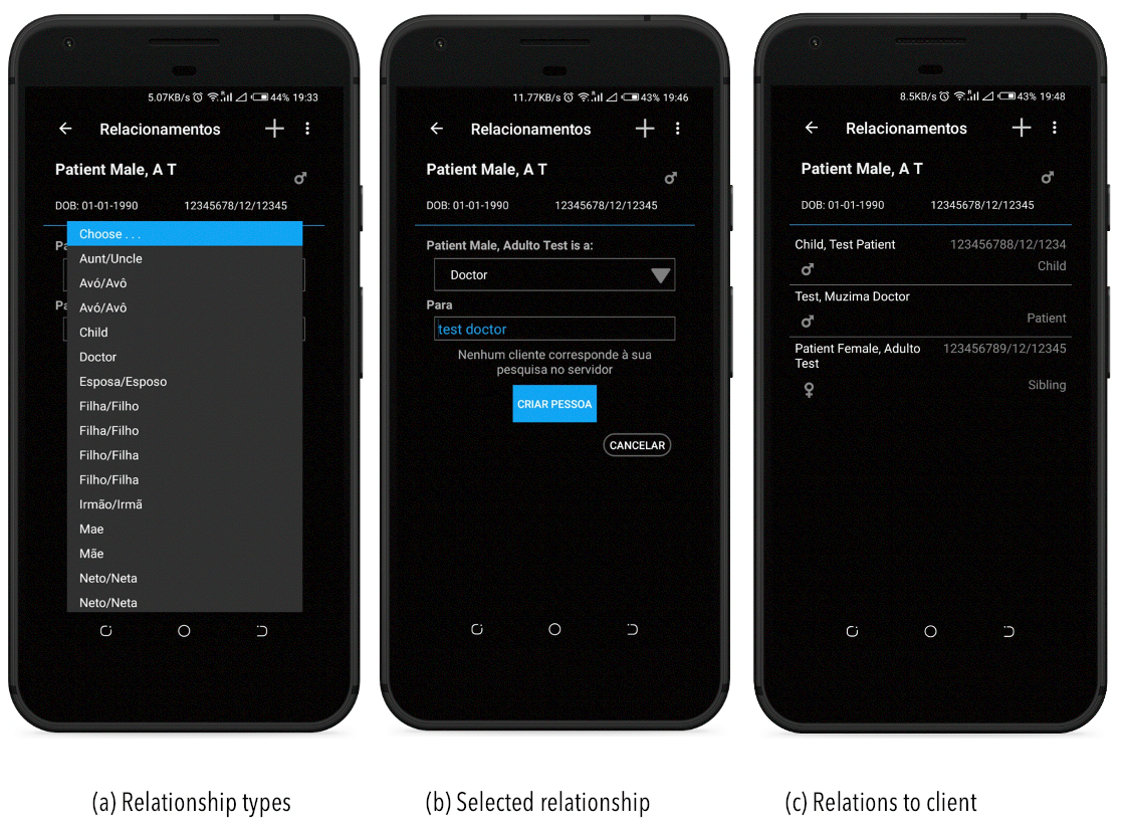

Supplement: Multimedia Appendix 3 [file jmir_v23i12e26381_app3.png]

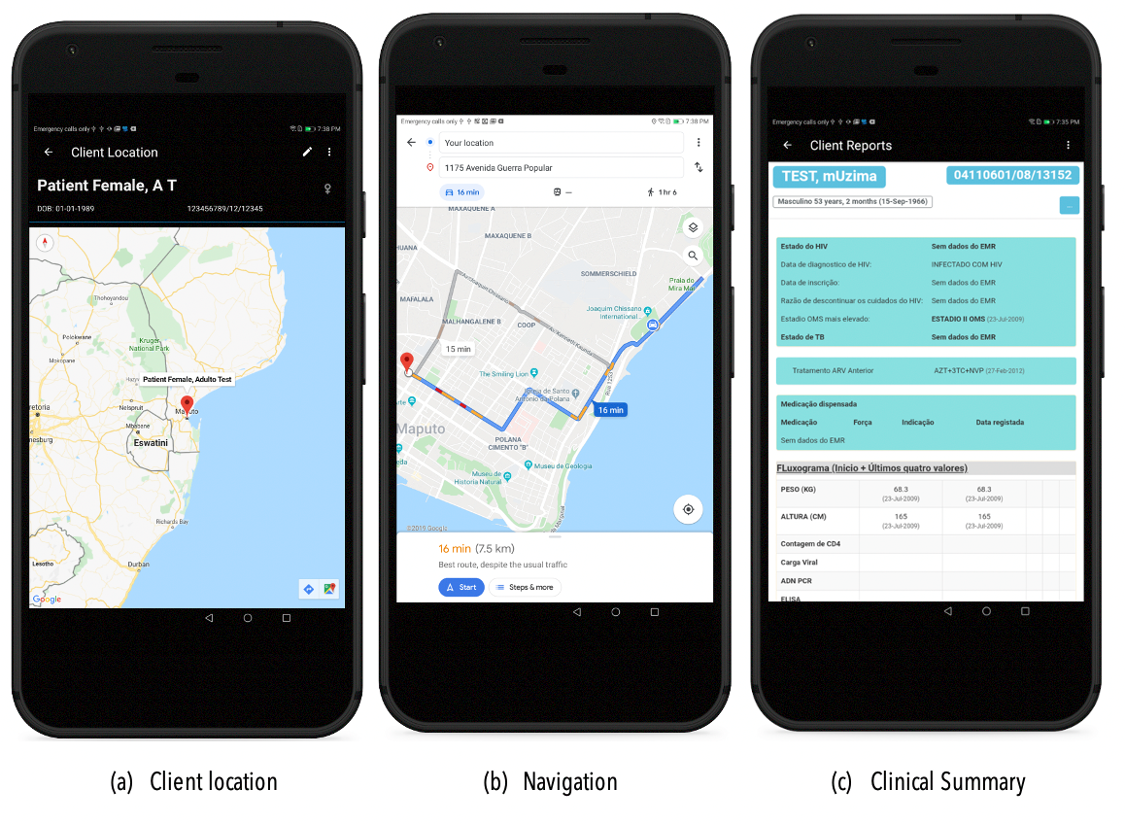

Supplement: Multimedia Appendix 4 [file jmir_v23i12e26381_app4.png]

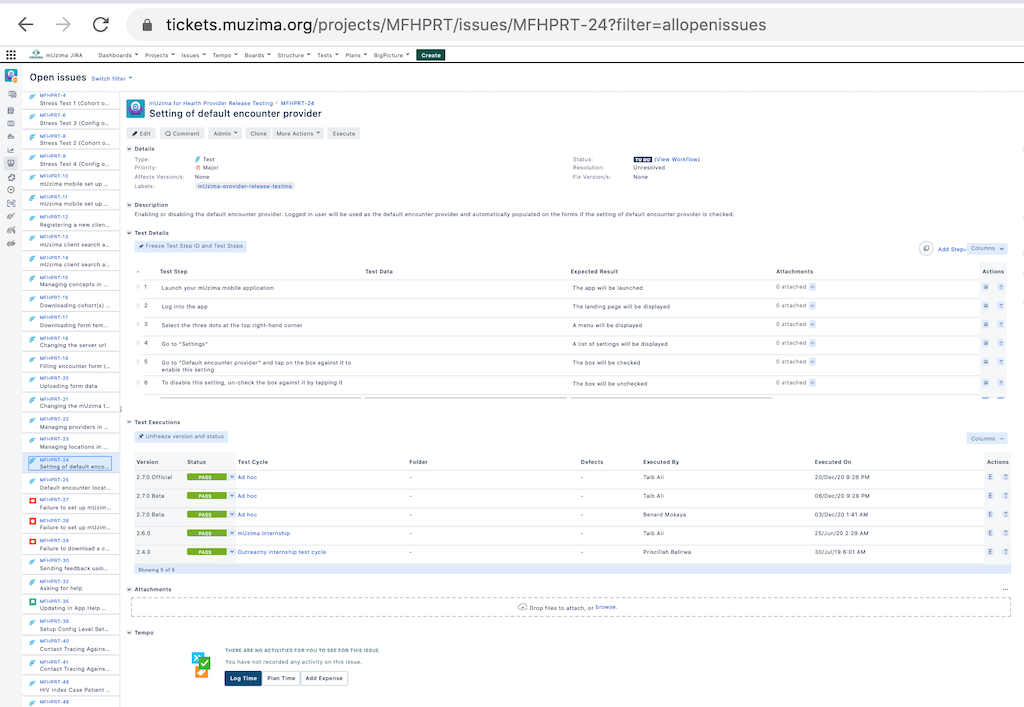

Supplement: Multimedia Appendix 5 [file jmir_v23i12e26381_app5.png]

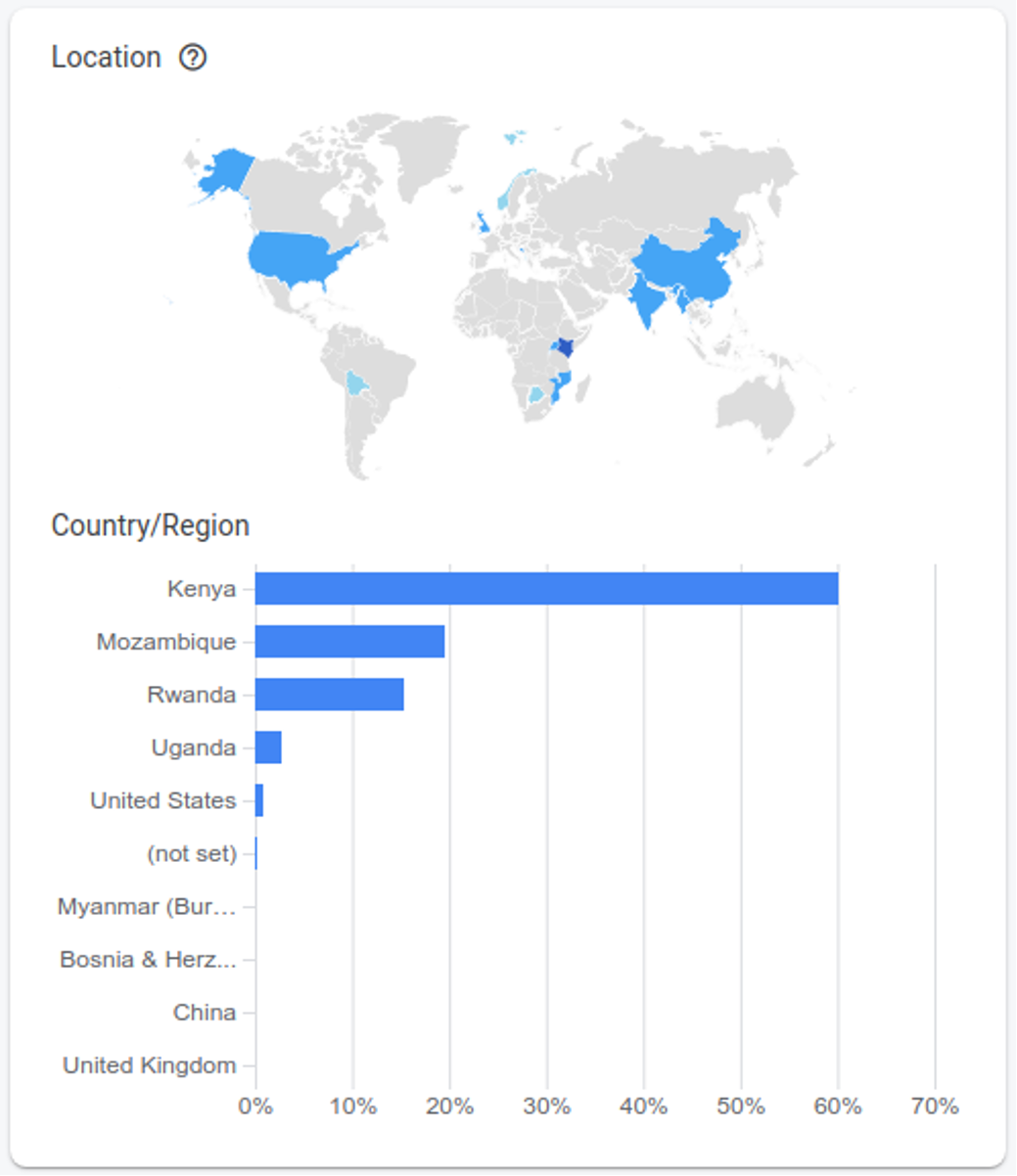

Supplement: Multimedia Appendix 8 [file jmir_v23i12e26381_app8.png]
